# Signature maps for automatic identification of prostate cancer from colorimetric analysis of H&E- and IHC-stained histopathological specimens

**DOI:** 10.1038/s41598-019-43486-y

**Published:** 2019-05-06

**Authors:** Ethan Leng, Jonathan C. Henriksen, Anthony E. Rizzardi, Jin Jin, Jung Who Nam, Benjamin M. Brassuer, Andrew D. Johnson, Nicholas P. Reder, Joseph S. Koopmeiners, Stephen C. Schmechel, Gregory J. Metzger

**Affiliations:** 1 Department of Biomedical Engineering, University of Minnesota, Minneapolis, MN USA; 2 Department of Lab Medicine and Pathology, University of Minnesota, Minneapolis, MN USA; 3 Department of Pathology, University of Washington, Seattle, WA USA; 4 Division of Biostatistics, School of Public Health, University of Minnesota, Minneapolis, Minnesota USA; 5 Department of Electrical and Computer Engineering, University of Minnesota, Minneapolis, MN USA; 6 Department of Radiology, Center for Magnetic Resonance Research, University of Minnesota, Minneapolis, MN USA

**Keywords:** Machine learning, Image processing, Prostate cancer, Biomedical engineering, Pathology

## Abstract

Prostate cancer (PCa) is a major cause of cancer death among men. The histopathological examination of post-surgical prostate specimens and manual annotation of PCa not only allow for detailed assessment of disease characteristics and extent, but also supply the ground truth for developing of computer-aided diagnosis (CAD) systems for PCa detection before definitive treatment. As manual cancer annotation is tedious and subjective, there have been a number of publications describing methods for automating the procedure via the analysis of digitized whole-slide images (WSIs). However, these studies have focused only on the analysis of WSIs stained with hematoxylin and eosin (H&E), even though there is additional information that could be obtained from immunohistochemical (IHC) staining. In this work, we propose a framework for automating the annotation of PCa that is based on automated colorimetric analysis of both H&E and IHC WSIs stained with a triple-antibody cocktail against high-molecular weight cytokeratin (HMWCK), p63, and α-methylacyl CoA racemase (AMACR). The analysis outputs were then used to train a regression model to estimate the distribution of cancerous epithelium within slides. The approach yielded an AUC of 0.951, sensitivity of 87.1%, and specificity of 90.7% as compared to slide-level annotations, and generalized well to cancers of all grades.

## Introduction

Prostate cancer (PCa) is the second most common cancer among men in the U.S^1^. While traditionally the diagnosis of PCa relies on the examination of prostate biopsy specimens, there is a wealth of clinically-significant information that can be gathered from assessment of prostate specimens obtained from radical prostatectomy (RP), which is one of the gold standards for treatment of localized PCa^2^. Assessment of RP specimens allows for the refinement of diagnoses made on biopsy specimens and the assessment of surgical margins and extraprostatic extension^3,4^, which in turn are used to determine the necessity of adjuvant therapies and to predict patient outcomes via nomograms^5–7^. PCa identified on RP specimens also serves as the ideal ground truth for the development of computer-aided diagnosis (CAD) systems^8^, which use, for example, information obtained from magnetic resonance imaging (MRI) to non-invasively predict the presence and extent of disease. However, accomplishing this requires the detailed examination of hematoxylin and eosin (H&E) stained sections of RP specimens by trained pathologists, which involves the manual annotation of PCa, i.e., the detection and delineation of cancerous tissue from benign tissue. This process is not only tedious and time-consuming, but also associated with significant inter-reader, experience-dependent variability^9,10^.

The recent advent of digital pathology and whole-slide imaging systems provides an opportunity to improve the pathology annotation process^11^. Stained sections digitized by whole-slide imaging systems at high resolution can be processed and analyzed by a variety of image analysis algorithms to extract and assess features such as stain intensity and nuclei density^12–14^, which relate to the likelihood of disease being present. These features can then be used to build a computational model to estimate the spatial distribution of disease on each whole-slide image (WSI), in effect automating the annotation process. In particular, deep learning techniques have been applied in recent years to digitized histopathologic images for the detection of a variety of cancers, including lung, prostate, breast, kidney, bladder, skin, and gastric cancer^15–20^. One limitation common to these works is that they rely solely on the analysis of H&E-stained slides for cancer detection. While H&E staining offers information about tissue morphology and architecture, it does not capture the gene expression profiles of the cells, which provides functional information that can inform disease likelihood. Therefore, image analysis of immunohistochemical (IHC) slides is a promising approach to extend and improve upon existing work.

Prostate adenocarcinoma, which comprises >90% of all prostate cancers, is histologically defined simply by the presence of glands without the outer basal cell layer. However, the accurate annotation of PCa is challenging. PCa tends to be locally infiltrative, and distinguishing malignant glands from surrounding benign glands can be tedious. The presence of the basal cell layer is often difficult to ascertain on H&E alone, which leads to underdiagnosis^21^. Additionally, there are several pathologic entities that are mimics of PCa. The most prominent of these is prostatic intraepithelial neoplasia (PIN). While PIN itself is considered benign, high-grade PIN (HGPIN) is suggestive of the presence of invasive carcinoma^22^. To further complicate matters, HGPIN is difficult to distinguish from intraductal carcinoma of the prostate (IDC-P), which is a malignant entity that usually represents the invasion of PCa into benign glands^23,24^. For these reasons, IHC is often used in aiding pathologic diagnosis of PCa. In particular, the triple-antibody cocktail specific for high-molecular weight cytokeratin (HMWCK), p63, and α-methylacyl CoA racemase (AMACR) is routinely used^25^. HMWCK and p63 are basal cell markers that act as negative cancer markers, i.e., the lack of immunoreactivity is indicative of the absence of the basal cell layer^21,26,27^. On the other hand, AMACR is a positive cancer marker that is usually highly overexpressed in PCa as well as HGPIN and IDC-P^21,28,29^. The combination of these three IHC markers has been shown to be superior for demonstrating PCa than any of them individually^25,30^.

Given that IHC staining for HMWCK + p63 + AMACR has a well-established role in aiding the histological diagnosis of PCa, we developed in this work methods for automated annotation of PCa on digitized whole slide images of prostatectomy specimens stained with H&E and the triple-antibody cocktail. Features were extracted from colorimetric image analysis of both H&E and IHC slides, and a regression model was trained to predict the extent and distribution of cancerous epithelium within each slide. The model was then applied to a large number of test cases, and the outputs were evaluated against slide-level manual annotation of PCa by pathologists.

## Methods

### Ethics statement

All experiments were approved under IRB protocol 0601M79888 with the University of Minnesota Institutional Review Board and carried out in accordance with approved guidelines. The IRB waived the need for informed consent for this retrospective analysis of de-identified samples.

### Patient cohort

A total of 184 prostate specimens were obtained from a cohort of 63 patients who underwent radical prostatectomy for definitive treatment of biopsy-proven prostate adenocarcinoma at our institution between November 2009 and January 2012. A summary of the patient characteristics is detailed in Table 1.

**Table 1:** Summary of the clinical and pathologic characteristics of the patient cohort.

Parameter	Data	
	Training set (n = 10)	Test set (n = 53)
Mean age (yrs)	61 (range: 55–72)	63 (range: 47–76)
Mean serum prostate specific antigen at time of surgery (ng/mL)	11.3 (range: 2.5–19.4)	7.85 (range: 0.40–37.60)
**Pathologic stage**		
T2a	0	9
T2b	0	4
T2c	4	26
T3a	5	10
T3b	1	4
**Gleason score**		
3 + 3	1	13
3 + 4	4	21
4 + 3	4	8
4 + 4	1	5
4 + 5	0	4
5 + 4	0	2

### Histopathology processing and staining

The prostate specimens were fixed and paraffin-embedded using a previously described protocol and sliced into 4 µm-thick axial sections^8,12^. From each tissue block, two sections were selected from tissue levels no more than 100 µm apart, and were de-paraffinized and rehydrated using standard methods. H&E and IHC staining was performed on the two sections, respectively. H&E staining was performed in three batches using routine clinical protocols. IHC staining was performed using a Ventana BenchMark ULTRA automated immunostainer platform (Ventana Medical Systems, Tucson, AZ). Antigen retrieval and blocking were performed as previously described^31^. Slides were incubated for 32 minutes with the triple-antibody cocktail containing primary antibodies to the basal cocktail of HMWCK + p63 (monoclonal mouse; clones 34βE12 and 4A4 respectively; prediluted; Ventana, Tucson, AZ) and AMACR (monoclonal rabbit; clone 13H4; prediluted; Dako, Glostrup, Denmark). Detection was performed with the Ventana ultraView Universal DAB Detection Kit and ultraView Universal Alkaline Phosphatase Red Detection Kit according to manufacturer’s instructions. This was followed by rinsing, counterstaining with hematoxylin, dehydrating, and coverslipping. In summary, HMWCK + p63 expression in benign basal epithelium was demonstrated as brown by 3,3-diaminobenzidine (DAB), AMACR expression in malignant epithelium was demonstrated as red by Fast Red chromogen, and stroma was demonstrated as blue by hematoxylin counterstain.

### Slide digitization and slide-level annotations

**Figure 1:**
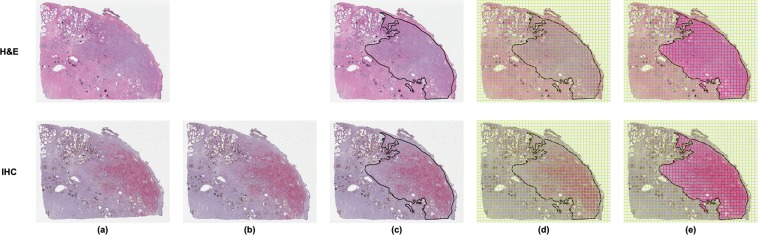
Use of SigMap software for initial processing of WSIs. This ensures the accurate spatial co-localization of H&E and IHC WSIs, and in turn the co-localization of image features extracted from both. (**a**) Digitized WSIs. (**b**) IHC WSI after rigid registration to the H&E WSI. (**c**) Regions of manually-annotated cancer outlined in black on the H&E WSI (GS 3 + 4 in this example). These regions were copied to the registered IHC WSI by SigMap. (**d**) Grid of analysis squares generated by SigMap overlaid on H&E and IHC WSIs. (**e**) Analysis squares with ≥75% overlap with the slide-level annotation identified by SigMap (in red). These analysis squares were subsequently labeled cancer and assigned the GS of the annotation.

Both H&E and IHC slides were digitized at 20x magnification (0.5 µm/pixel) using a whole slide scanner (Aperio ScanScope CS, Leica Biosystems, Buffalo Grove, IL). Digitized H&E WSIs were annotated at the slide-level for PCa by pathology trainees (B.M.B., A.D.J., N.P.R.) under the supervision of a board-certified pathologist (S.C.S.) using Aperio’s ImageScope software (Leica Biosystems, Buffalo Grove, IL) and a pen-tablet screen (Wacom Cintiq 22HD, Saitama, Japan). The *slide-level annotations* were carried out by demarcating the borders of distinct regions of cancer and assigning a Gleason score (GS) to each region (Fig. 1c). Using the same tools, *negative annotations*, defined as regions containing artifacts of the histological processing (e.g., tissue folds, debris, irregular staining), were demarcated on the IHC WSIs by technologists (A.E.R., J.C.H.). Regions of negative annotations were ultimately excluded from analysis, and typically comprised no more than 5% of a given slide. Digitized WSIs and annotations were stored and managed as previously described^32^.

SigMap software was used to further process the digitized WSIs^12,33^. First, it was used to register the IHC WSI to the H&E WSI using a rigid transformation (Fig. 1a,b). Next, binary masks of the slide-level cancer annotations and the negative annotations were created to transfer the annotations between H&E WSIs and IHC WSIs (Fig. 1c). A virtual grid composed of analysis squares (dimensions 1,000 × 1,000 pixels, area of 0.25 mm^2^) was then generated by SigMap and added to both WSIs (Fig. 1d). Analysis squares whose areas overlapped at least 75% with the cancer annotation mask were labeled as cancer and assigned the GS of the corresponding annotation (Fig. 1e). Analysis squares whose areas overlapped at least 75% with the negative annotation mask were excluded from further analysis.

### Colorimetric image analysis algorithms

The following three quantitative image analysis algorithms (Aperio Brightfield Image Analysis Toolbox, Leica Biosystems, Buffalo Grove, IL) were configured by a technologist (J.C.H.), then applied to H&E and IHC WSIs in order to extract features for prediction of cancer.

The Positive Pixel Count (PPC) algorithm was applied to H&E WSIs. Briefly, the PPC algorithm counts the number of stained pixels within each analysis square that falls within and out of a specified range of hue-saturation-brightness (HSB) color values (*positive* and *negative pixels*, respectively). HSB values were sampled from three types of regions that predominantly contained a single histological feature of interest (nuclei, cytoplasm, or stroma). Fifteen of each type of region were manually identified on control H&E WSIs and sampled. Ranges of HSB values were calculated for each type of region and were manually adjusted to eliminate overlap between ranges. A separate PPC algorithm was configured for each type of region and its corresponding range of HSB values. The three configured PPC algorithms were then applied prospectively to analysis squares of H&E WSIs. The resulting numbers of positive pixels were converted to percentages of the analysis square occupied by nuclei, cytoplasm, and stroma (% nuclei, % cytoplasm, and % stroma, respectively), which were in turn used as predictive features. The unstained percentage of each analysis square was also calculated as % unstained = 100%−(% nuclei + % cytoplasm + % stroma), and analysis squares with % unstained >99% were excluded from further analysis on the basis that they are taken from regions outside of the tissue boundaries. To account for variations in H&E staining intensity across the three batches, a different set of PPC algorithms was configured and applied to each batch.

Color Deconvolution (CD) and Co-expression (CE) algorithms were applied to IHC WSIs to measure the colorimetric features of the IHC stain. Briefly, the CD algorithm isolates individual staining components of IHC WSIs for quantification, while the CE algorithm quantifies how often the staining components occur separately and together. These algorithms were first configured on control slides. Three control slides were cut, processed, and singly-stained with either DAB chromogen (brown), Fast Red chromogen (red), or hematoxylin counterstain (blue), using the same protocols as the triple-stained IHC slides described above. The average red-green-blue (RGB) optical density (OD) values of the three components were sampled from the corresponding WSIs of the control slides and were measured as Fast Red (R: 0.283, G: 0.949, B: 0.757), DAB (R: 0.461, G: 0.826, B: 1.0), and hematoxylin (R: 0.21, G: 0.276, B: 0.176), and intensity thresholds were manually configured for each component to define positively-stained pixels. The configured CD and CE algorithms were then applied prospectively to analysis squares of IHC WSIs, from which the percentage of each analysis square that was positively staining (%Pos) was calculated. As previously described, the OD quantifies the stain intensity, as it is linearly related to the amount of staining^13,31,32,34^.

Using the configured RGB OD and intensity threshold values, IHC WSIs were then separated into brown, red, and blue color channels corresponding to each staining component. The brown and red staining were separately quantified by the CD algorithm as previously described^32^. Specifically, the average OD and %Pos were measured by the CD algorithm for both brown and red components, and the products OD × %Pos were calculated and used as predictive features. The co-localization of brown and red staining was quantified by the CE algorithm, which was then used to calculate the percentage of the analysis square that was positively staining for only red or only brown, but not both (%Pos_CE_). %Pos_CE_ for red and brown components were used as predictive features.

In summary, seven features were extracted from each analysis square (Table 2). The features derived from H&E WSIs were the percentages of nuclei, cytoplasm, and stroma, while the features derived from IHC WSIs were the percentages and stain intensities (quantified by the OD) of brown and red staining, which corresponded to the characteristics of the basal cell staining (HMWCK + p63) and the AMACR staining, respectively.

**Table 2:** Summary of the extracted features. Features are calculated on an analysis-square level.

Feature	Source	Algorithm
% Nuclei	H&E	Positive Pixel Count (nuclear)
% Cytoplasm	H&E	Positive Pixel Count (cytoplasmic)
% Stroma	H&E	Positive Pixel Count (stromal)
OD × %Pos (brown)	IHC	Color Deconvolution (brown)
OD × %Pos (red)	IHC	Color Deconvolution (red)
%Pos_CE_ (brown)	IHC	Co-expression
%Pos_CE_ (red)	IHC	Co-expression

### Training data and analysis square-level annotations

**Figure 2:**
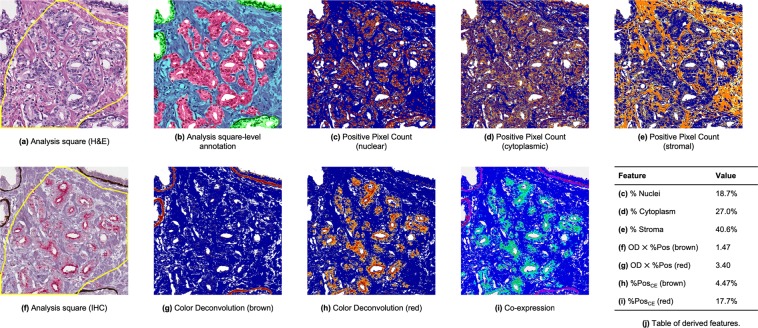
Examples of pseudo-color outputs of Aperio image analysis algorithms from which the predictive features were calculated. (**a**) Analysis square from an H&E WSI in the training set (75% overlap with the slide-level annotated cancer outlined in yellow). (**b**) Analysis square-level annotation of (**a**), with benign epithelium in green, malignant epithelium in red, gland lumens in white, and stroma in blue. The percentage of malignant epithelium is used as the ground truth for training. (**c–e**) Outputs of the PPC algorithms. Positive pixels are in red/orange/yellow, and negative pixels in blue. The percentages of positive pixels is taken to be the percentages of the analysis square occupied by nuclei, cytoplasm, or stroma. (**f**) Analysis square corresponding to (**a**) taken from the corresponding IHC WSI. (**g**,**h**) Outputs of the CD algorithms. Positive pixels are in yellow/orange/red, and negative pixels in blue. (**i**) Output of the CE algorithm. Positive pixels for red and brown components are in green-cyan and red-purple, respectively. (**j**) Table of features derived from the outputs of the image analysis algorithms.

Ten of the 63 patients in our cohort were randomly selected, and one pair of WSIs was created from each for purposes of training the regression model. Forty analysis squares were randomly selected from each of the ten pairs of WSIs (400 analysis squares in total) and were manually annotated in much greater detail than usual (S.C.S.). The *analysis square-level annotations* were carried out by meticulously delineating the benign and cancerous epithelium, gland lumens, stroma, and regions of clear glass within the 1,000 × 1,000 pixel-area of each analysis square. The fractional areas of each of the aforementioned components were then summated for each annotated analysis square (Fig. 2b). The percentage of cancerous epithelium within each analysis square-level annotation was taken to be the ground truth. Details on the slides can be found in Table 3.

**Table 3:** Breakdown of the distribution of the analysis squares of the training and test data by cancer presence and Gleason score.

Type	Training (10 total)	Test (174 total)
Cancer	84	23,757
3 + 3	13 (1)	2,849 (31)
3 + 4	37 (4)	6,146 (47)
4 + 3	34 (4)	4,452 (22)
4 + 4	0 (0)	2,790 (15)
4 + 5	0 (0)	6,146 (16)
5 + 4	0 (0)	1,374 (4)
**Benign**	**316**	**189,629**
**Totals**	**400**	**213,386**

An analysis square was labeled cancer if it overlapped at least 75% with the slide-level annotation. Excluded analysis squares (i.e., those that overlapped at least 75% with the negative annotation, or were found to be >99% unstained on H&E staining) are not tabulated here. Numbers in parentheses indicate the number of pairs of WSIs containing cancer with the corresponding Gleason score. Note that some WSIs contained no annotated cancer (1 in the training set, 39 in the test set).

### Regression model training and evaluation

Elastic net regression models were trained on these data using 10-fold cross validation^35^, with each fold containing the 40 analysis squares from a single pair of WSIs. The elastic net is a generalized linear regression model with both L1 and L2 regularization, and its corresponding objective function to be minimized is

$$\mathop{\min }\limits_{\omega }\frac{1}{2m}{\Vert X\omega -y\Vert }_{2}^{2}+\alpha \rho {\Vert \omega \Vert }_{1}+\frac{\alpha (1-\rho )}{2}{\Vert \omega \Vert }_{2}^{2}$$

where *m* is the number of training examples, *n* is the number of features, *X* is the *m*-by-*n* matrix of training examples, *ω* is the *n*-by-1 vector of feature weights, *y* is the *m*-by-1 vector of labels, and *α* and *ρ* are parameters that determine the strengths of the regularization terms.

Models were trained on four different sets of features: (1) features from H&E WSIs alone (the *H&E model*), (2) features from IHC WSIs alone (the *IHC model*), (3) features from both H&E and IHC WSIs, but without the two %Pos_CE_ features (the *full*_–*CE*_ *model*), and (4) all features from both H&E and IHC WSIs (the *full model*). Given the similarity of %Pos from the CD algorithm and %Pos_CE_ from the CE algorithm, both the full_–CE_ model and the full model were included in order to test if the inclusion of the two %Pos_CE_ features would provide any benefit to cancer identification accuracy.

For each model, the coefficients of the two regularization terms (*α* and *ρ*) were treated as hyperparameters and selected by cross-validation to minimize the mean value of the objective function (averaged across the ten folds). Trained models were then applied to the analysis squares of the other 174 pairs of slides to produce predicted maps of cancerous epithelium. Model outputs were compared to the slide-level annotations on a per-analysis square level using receiver operating characteristic (ROC) curve analysis. Sensitivities and specificities were calculated using the optimum cut-off points for the ROC curves that corresponded to the maxima of the Youden indices. The 95% confidence intervals (CIs) were calculated using a bootstrap procedure that resampled both WSIs and analysis squares from the training set, and only WSIs from the test set. Two-sided p-values were found by inverting the 95% bootstrap CIs.

## Results

### Outputs of colorimetric image analysis algorithms

Figure 2a,f illustrate the detail at the level of an analysis square for H&E and IHC WSIs, respectively. Figure 2c–e show sample outputs of the PPC algorithms. Figure 2g,h show sample outputs of the CD and CE algorithms, respectively. As the degree of co-localization between the brown and red components of the deconvolved IHC slides is generally small (typically <5% of the analysis square), the overlap is difficult to visualize in Fig. 2i.

### Quantitative evaluation of model performance

**Figure 3:**
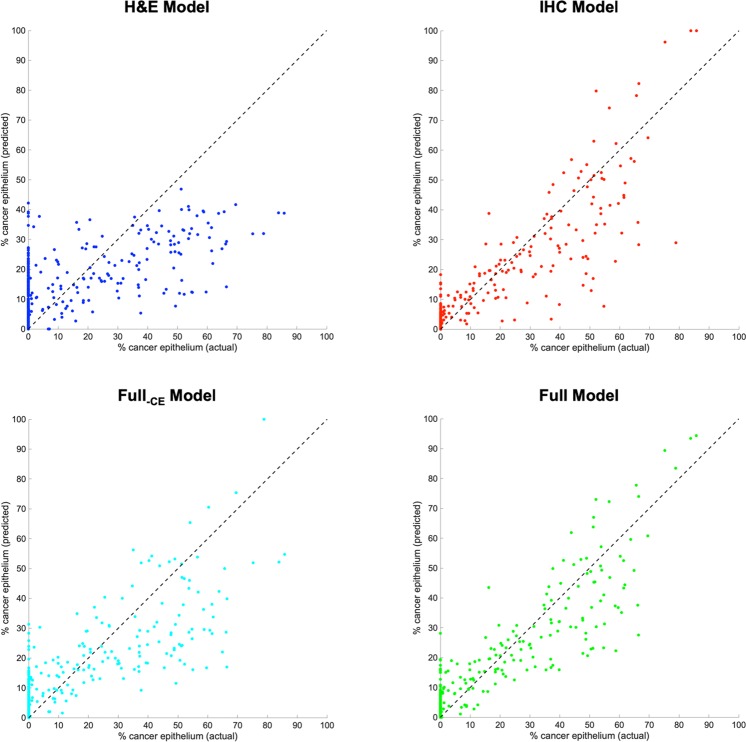
Cross-validation scatterplots of the predicted vs. actual % cancer epithelium for the four regression models trained with different feature sets. Data points in each plot were accumulated across the ten cross-validation folds.

Cross-validation performance for the four models was evaluated by plotting cumulative scatterplots of predicted % cancer epithelium vs. the actual % cancer epithelium across the 10 cross-validation folds (Fig. 3). The cross-validation root mean square error, median absolute error, and maximum absolute error for each model are shown in Table 4.

**Table 4:** Comparison of the cross-validation performance for the four regression models.

Model	Root mean square error	Median absolute error	Maximum absolute error
H&E model	15.4	8.37	52.3
IHC model	9.36	3.55	49.9
Full_–CE_ model	11.9	5.59	49.4
Full model	8.37	3.09	38.8

**Figure 4:**
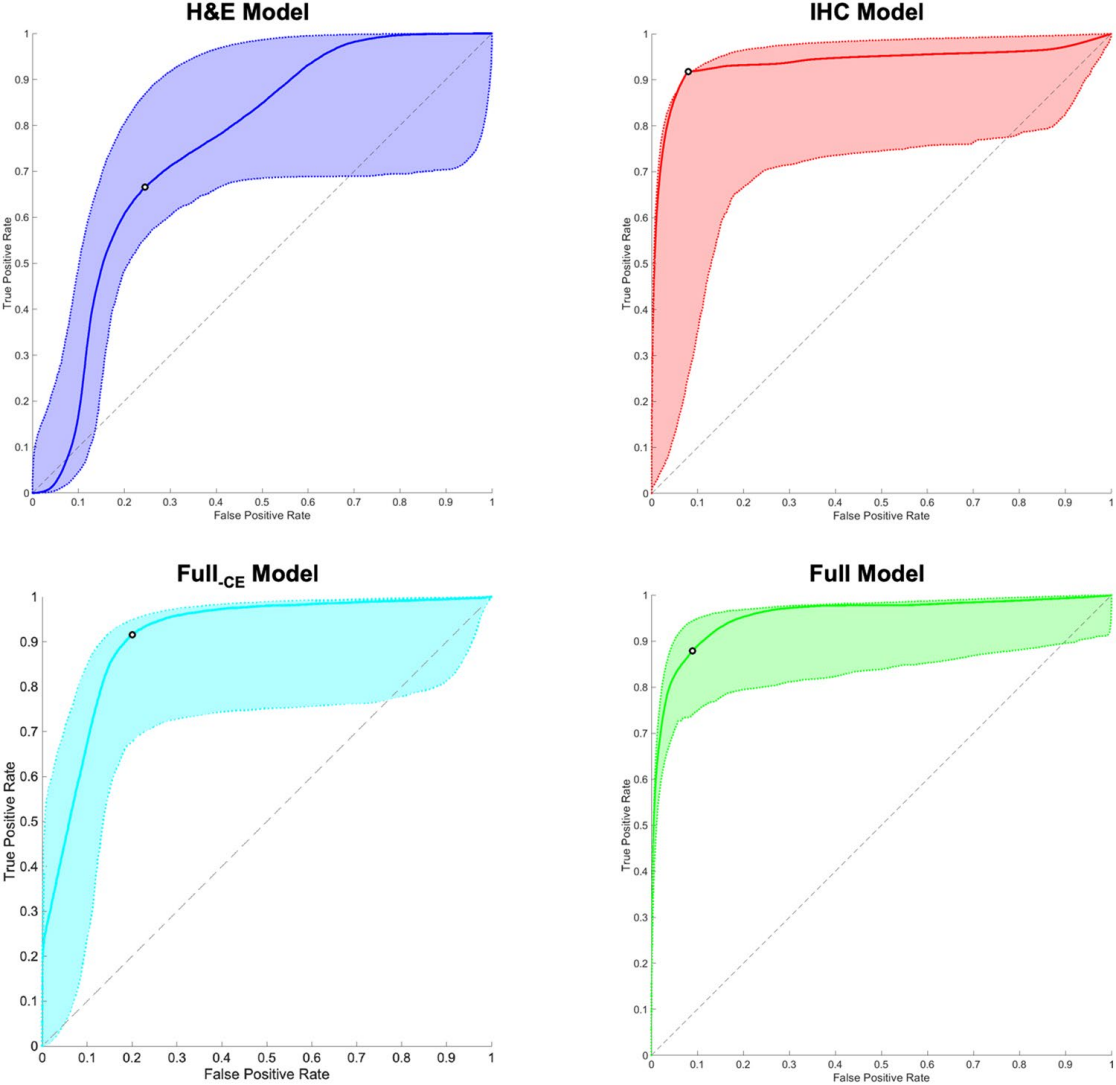
Receiver operating characteristic (ROC) curves for the regression models trained with different feature sets. Shaded regions correspond to the 95% bootstrap confidence intervals generated from 1,000 bootstrap samples. Black circles indicate the maxima of the Youden indices, which were chosen as the cutoff points.

Performance on the test set for the four models was evaluated by plotting the ROC curves (Fig. 4). The area under the ROC curves (AUCs), as well as the sensitivities and specificities calculated at the respective maxima of the Youden indices, are shown in Table 5. The AUC for the full model was significantly higher than that of the H&E model (p = 0.026), while the specificity for the full model was significantly higher than those of the H&E and full_–CE_ models (p < 0.001 for both). The AUC and specificity for the full model were not significantly different than those of the IHC model (p = 0.542 and p = 0.108, respectively). The sensitivity of the full model was also not significantly different than those of the H&E, IHC, and full_–CE_ models (p = 0.134, p = 0.748, and p = 0.939, respectively). The CIs of these summary statistics for the full model were notably narrower than those of the other three models, suggesting that the performance of the full model will likely be closer to what is reported here when it is applied prospectively.

**Table 5:** Comparison of classification performance for the four regression models. Numbers in brackets are the 95% bootstrap confidence intervals generated from 1,000 bootstrap samples.

Model	AUC	Sensitivity	Specificity
H&E model	0.755 [0.582, 0.867]	0.661 [0.562, 0.898]	0.760 [0.665, 0.803]
IHC model	0.937 [0.692, 0.961]	0.918 [0.661, 0.931]	0.920^†^ [0.780, 0.938]
Full_–CE_ model	0.911 [0.682, 0.943]	0.907 [0.683, 0.924]	0.809 [0.765, 0.864]
Full model	0.951^†^ [0.832, 0.964]	0.871 [0.753, 0.934]	0.907^†,*^ [0.894, 0.959]

^†^Significant at p < 0.05 compared to the H&E model.

^*^Significant at p < 0.05 compared to the full_–CE_ model.

For the full model, the sensitivity of PCa detection was broken down by both Gleason score and Gleason grade group (GG)^36^, the latter of which may better reflect cancer aggressiveness (Table 6). Using the convention that GG ≤ 2 (GS 3 + 3 or 3 + 4) is low to intermediate-grade and GG ≥ 3 (GS 4 + 3, 4 + 4, 4 + 5, or 5 + 4) is high-grade, the sensitivity of detecting low to intermediate-grade cancers was 0.884, while it was 0.864 for high-grade cancers; this difference was found to be not significant (p = 0.107).

**Table 6:** Sensitivity of the full model broken down by Gleason score and Gleason grade groups.

Type	Number of Analysis Squares	Number Correctly Labeled	Sensitivity
3 + 3	2,849	2,411	0.846 [0.784, 0.957]
3 + 4	6,146	5,539	0.901 [0.721, 0.954]
GG ≤ 2	8,995	7,950	0.884 [0.792, 0.971]
4 + 3	4,452	4,098	0.921 [0.788, 0.956]
4 + 4	2,790	2,246	0.805 [0.732, 0.976]
4 + 5	6,146	5,135	0.836 [0.601, 0.891]
5 + 4	1,374	1,274	0.927 [0.715, 1]
GG ≥ 3	14,762	12,753	0.864 [0.727, 0.934]
Totals	23,757	20,703	0.871 [0.742, 0.929]

Low to intermediate-grade cancers were defined by GG ≤ 2 (GS = 3 + 3 or 3 + 4), and high-grade cancers were defined by GG ≥ 3 (GS = 4 + 3, 4 + 4, 4 + 5, or 5 + 4). Numbers in brackets are the 95% bootstrap confidence intervals generated from 1,000 bootstrap samples.

### Comparison of model-generated annotations to manual slide-level annotations

**Figure 5:**
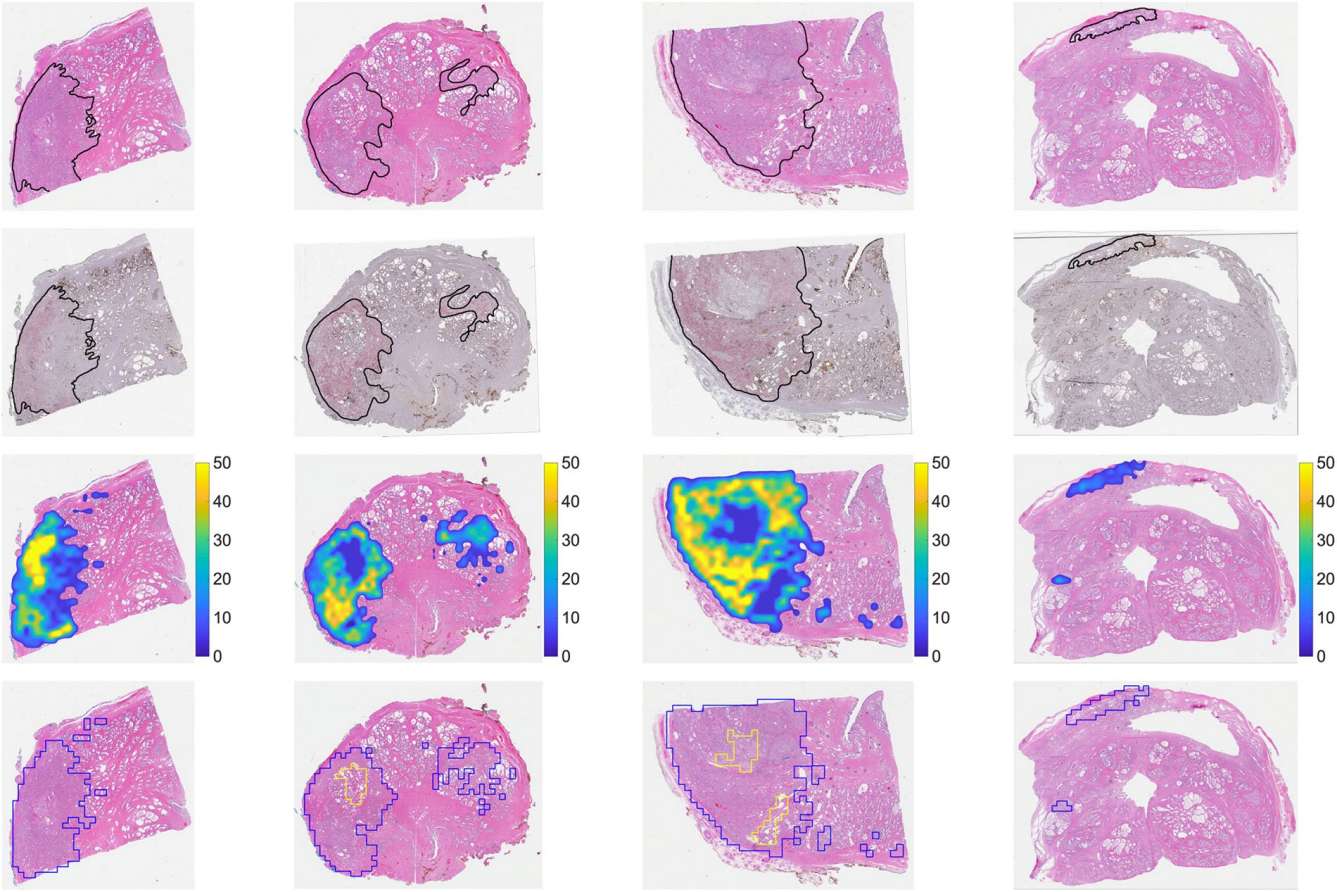
Representative comparisons of slide-level annotations to model-generated prediction maps. **Row 1**: H&E WSIs with slide-level annotations outlined in black. **Row 2**: IHC WSIs corresponding to the H&E WSIs in Row 1. Sigmap software was used to perform registration to the H&E WSIs, and to copy the annotated cancer. **Row 3**: Model-generated maps of the predicted distribution of malignant epithelium overlaid on the H&E WSIs. Colorbars correspond to the percentage of malignant epithelium. **Row 4**: Thresholded versions of prediction maps shown in Row 3, with the Youden index (2.61%) chosen as the threshold. Predicted slide-level annotations are outlined in blue, with internal benign regions outlined in yellow.

Figure 5 shows representative H&E and IHC slides with slide-level, manually-annotated cancer by pathologists compared with maps generated by the full model. Note the high degree of correlation between the annotated cancer, distribution of AMACR staining (red on IHC slides), and predicted distribution of malignant epithelium.

## Discussion

In this work, we show that a predictive model that uses features derived from colorimetric analysis of both digitized H&E and IHC slides is able to detect and delineate PCa on WSIs with accuracy comparable to pathologists’ slide-level annotations. The performance of the full model was found to be superior to those of the other three models, individually (Table 5), though this difference was only significant in comparison to the H&E model. Furthermore, despite the relatively small amount of training data that included predominantly low and intermediate-grade cancers, the model performed well across a large number of test set slides. Its sensitivity was also largely consistent across cancers with different Gleason scores, and was only slightly worse for high-grade cancers (0.864 vs. 0.871 for all cancers, Table 6).

In contrast to most published works, the regression model described here uses a compact set of seven features that were calculated from the outputs of standard image analysis algorithms applied to H&E WSIs (% nuclei, % cytoplasm, % stroma) and IHC WSIs (OD × %Pos and %Pos_CE_, for brown and red). These features were ultimately chosen for their simplicity and interpretability. Although %Pos from the CD algorithm and %Pos_CE_ from the CE algorithm appeared to be redundant, the results demonstrate that excluding the two %Pos_CE_ features resulted in worse specificity of cancer detection (full_−CE_ model vs. full model, Table 5). This is most likely due the fact that some slides contained a larger fraction of non-cellular components (e.g., intraglandular cellular debris, corpora amylacea) that stained both brown and red with IHC staining. This would cause %Pos (red) as calculated by the CD algorithm to be falsely elevated, but would not affect %Pos_CE_ (red) as calculated by the CE algorithm as the CE algorithm excludes regions that stained both brown and red. Therefore, inclusion of the two %Pos_CE_ features increased the specificity of cancer detection for slides containing a significant fraction of such regions, and in turn increased the overall specificity of cancer detection.

Another notable aspect of the work is that the full model was trained to predict the percentage of cancerous epithelium, which was made possible by the unique ground truth obtained from the meticulous annotation of individual analysis squares. For purposes of model training, this ground truth is superior to the slide-level annotations, as those are known to have finite accuracy and precision^9,10^.

**Figure 6:**
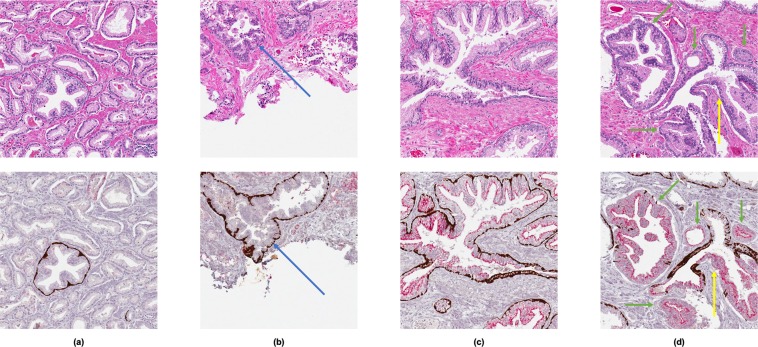
Illustrative examples of analysis squares with discrepancies between the slide-level annotation and the model output. The top row shows H&E analysis squares, and the bottom row shows the corresponding IHC analysis squares. (**a**) Analysis square with predominantly malignant glands that have poor AMACR staining. 100% overlap with the slide-level annotation, but incorrectly labeled as non-PCa by the model. (**b**) Analysis square with PIN that has poor AMACR staining (blue arrow) and a large cystic region (bottom half). Correctly labeled non-PCa by the model, but had 100% overlap with slide-level annotation. (**c**) Analysis square with PIN that has strong AMACR staining. Did not overlap with the slide-level annotation, but was incorrectly labeled as PCa by the model. (**d**) Analysis square with small malignant glands (green arrows), including an example of HGPIN/IDC-P (yellow arrows). Correctly labeled PCa by the model, but did not overlap with the slide-level annotation.

The trained models were evaluated against the slide-level annotations on a per-analysis square level using ROC curve analysis. However, despite the high AUC achieved by the full model, it is difficult to assess the true performance of the model due again to the limitations of the slide-level annotations. Accurate assessment would require analysis square-level annotations of all the slides in the test set, which would be prohibitive. Qualitatively, visual comparison of the model-generated maps of cancerous epithelium with the slide-level annotations shows generally good concordance (Fig. 5). Sources of disagreements between the two can be divided into four categories, which are illustrated in Fig. 6:

1. Cancer missed by the model (Fig. 6a). This was most often due to cancer with poor AMACR staining; while AMACR is a sensitive positive marker of PCa, it is well-documented that some variants of PCa do not exhibit increased expression of AMACR^29,30,37,38^. Alternatively, inconsistencies in the staining procedure may have caused variabilities in AMACR staining, and these variabilities would be amplified in regions of cancer.
2. Cancer incorrectly annotated by the pathologist (Fig. 6b). This was most often due to large regions of glass (e.g., cystic areas, luminal areas of malignant glands) that are looped in with the slide-level annotations. More rarely, benign glands were incorrectly annotated as cancer; usually, these were examples of PIN with low AMACR expression.
3. Cancer incorrectly labeled by the model (Fig. 6c). This was most often due to PIN with high AMACR expression.
4. Cancer missed by the pathologist (Fig. 6d). This was most often due to small, isolated regions of cancer that were not annotated. More rarely, HGPIN and/or glands with IDC-P were missed by the pathologist but identified as cancer by the model due to high AMACR expression.

In summary, accurately distinguishing the different possible presentations of PCa from PIN is a challenge for both pathologists and the full predictive model. Although in theory glands with PIN are characterized by the presence of an intact basal cell layer, the basal cell layer may be quite fragmented, which would make it difficult to assess by either visual inspection or by quantitative assessment of brown staining intensity.

There are three major limitations of the features. First, since they are calculated from colorimetric analysis of stained WSIs, their consistency is highly-dependent on the reproducibility of the staining procedure and digitization process. As noted in previous works, the use of different histology protocols and/or different slide scanners can cause large variations in the morphological features of WSIs, which in turn degrade the predictive performance of trained models^20^. In our work, the three separate batches of H&E staining presented a major source of potential variability in the calculated H&E features, as the stain intensities were visibly different between H&E WSIs of different batches. To compensate, a different set of PPC algorithms was configured for each batch, though this was not ideal. In order to minimize batch effects in the future, H&E staining will also be performed on an automated platform using a standardized protocol, like what was done for the IHC staining. Additionally, algorithms like the PPC algorithm that rely on the analysis of intensity values (e.g., RGB or HSB values) are naturally quite prone to variations in stain intensity. Therefore, it would be worth extending the use of color deconvolution algorithms for the analysis of H&E WSIs, as proposed in previous works^39^. To expand this work to larger datasets, further methods for normalization of WSIs and/or derived features may also be investigated^20,39^.

Another limitation of the features is that they are derived from WSIs of different tissue levels of the tissue block. Due to technological constraints, H&E and IHC staining were not performed on the same tissue section, and thus two sections were taken from each specimen. Although the sections were spatially adjacent (separated by ≤100 µm), there were sometimes noticeable differences between the two when digitized and viewed at the magnification level of individual analysis squares (Figs 2a,f, 6b). However, these differences were relatively minor and unlikely to significantly affect the calculated features, and can further be minimized in the future by always selecting serial levels of the tissue block for staining. Methods for visualizing multiple stains on the same tissue section may also be considered^40,41^.

Lastly, the features only characterize the composition within each analysis square, and not the arrangement (i.e., cellular architecture) of the components. Therefore, the predictive model has difficulties distinguishing between analysis squares containing PIN and those containing a mixture of benign and malignant glands. This limitation may be addressed in future work by identifying additional IHC markers that are differentially expressed in cancer and PIN; for example, IDC-P is characterized by decreased expression of PTEN, which can be used to distinguish HGPIN from IDC-P^42^. An alternative approach could be to develop custom algorithms for object detection and segmentation (e.g., for identification of whole prostate glands). A more straightforward approach could be to supplement the training data with examples of PIN or PIN-like entities. Augmenting the training data may also allow the use of deep learning approaches such as convolutional neural networks^15–19^ that can learn features that account for differences in the glandular architecture within analysis squares.

In summary, the methods introduced in this work can be modularly integrated into digital pathology frameworks for detection of prostate cancer on whole-slide images of histopathology slides. The unique aspect of this work is that it incorporates information from slides with conventional H&E staining as well as those with IHC staining, and as demonstrated in this work, the combination of both allows for more accurate identification of prostate cancer. Given the number of previously identified and characterized genetic markers in other types of cancers, the methods presented here may be extended naturally to other types of cancer as well.

## Data Availability

The processed digitized pathology data, the outputs of the colorimetric analysis algorithms and the regression model, and the statistical analyses for the current study are available from the corresponding author upon reasonable request.

## References

[1] Siegel RL, Miller KD, Jemal A (2017). Cancer Statistics, 2017. CA: a cancer journal for clinicians.

[2] Heidenreich A (2014). EAU guidelines on prostate cancer. part 1: screening, diagnosis, and local treatment with curative intent-update 2013. European urology.

[3] Swindle P (2008). Do margins matter? The prognostic significance of positive surgical margins in radical prostatectomy specimens. The Journal of urology.

[4] McNeal JE, Villers AA, Redwine EA, Freiha FS, Stamey TA (1990). Capsular penetration in prostate cancer. Significance for natural history and treatment. The American journal of surgical pathology.

[5] Lughezzani G (2014). Multicenter European external validation of a prostate health index-based nomogram for predicting prostate cancer at extended biopsy. European urology.

[6] Brockman JA (2015). Nomogram Predicting Prostate Cancer-specific Mortality for Men with Biochemical Recurrence After Radical Prostatectomy. European urology.

[7] Stephenson AJ (2005). Postoperative Nomogram Predicting the 10-Year Probability of Prostate Cancer Recurrence After Radical Prostatectomy. Journal of clinical oncology: official journal of the American Society of Clinical Oncology.

[8] Metzger GJ (2016). Detection of Prostate Cancer: Quantitative Multiparametric MR Imaging Models Developed Using Registered Correlative Histopathology. Radiology.

[9] Allam CK (1996). Interobserver variability in the diagnosis of high-grade prostatic intraepithelial neoplasia and adenocarcinoma. Mod Pathol.

[10] Montironi R (2005). Gleason grading of prostate cancer in needle biopsies or radical prostatectomy specimens: contemporary approach, current clinical significance and sources of pathology discrepancies. BJU international.

[11] Gurcan MN (2009). Histopathological Image Analysis: A Review. IEEE reviews in biomedical engineering.

[12] Metzger GJ (2012). Development of multigene expression signature maps at the protein level from digitized immunohistochemistry slides. PloS one.

[13] Krajewska M (2009). Image Analysis Algorithms for Immunohistochemical Assessment of Cell Death Events and Fibrosis in Tissue Sections. Journal of Histochemistry and Cytochemistry.

[14] Kather JN (2016). Multi-class texture analysis in colorectal cancer histology. Scientific Reports.

[15] Cruz-Roa, A. *et al*. Accurate and reproducible invasive breast cancer detection in whole-slide images: A Deep Learning approach for quantifying tumor extent. *Scientific Reports* **7**, 10.1038/srep46450 (2017). 10.1038/srep46450 PMC5394452 28418027

[16] Arevalo J, Cruz-Roa A, Arias V, Romero E, Gonzalez FA (2015). An unsupervised feature learning framework for basal cell carcinoma image analysis. Artificial intelligence in medicine.

[17] Sharma H, Zerbe N, Klempert I, Hellwich O, Hufnagl P (2017). Deep convolutional neural networks for automatic classification of gastric carcinoma using whole slide images in digital histopathology. Computerized medical imaging and graphics: the official journal of the Computerized Medical Imaging Society.

[18] Litjens G (2016). Deep learning as a tool for increased accuracy and efficiency of histopathological diagnosis. Scientific Reports.

[19] Khosravi P, Kazemi E, Imielinski M, Elemento O, Hajirasouliha I (2018). Deep Convolutional Neural Networks Enable Discrimination of Heterogeneous Digital Pathology Images. EBioMedicine.

[20] Kothari S (2014). Removing batch effects from histopathological images for enhanced cancer diagnosis. IEEE journal of biomedical and health informatics.

[21] Humphrey PA (2007). Diagnosis of adenocarcinoma in prostate needle biopsy tissue. Journal of Clinical Pathology.

[22] Montironi R, Mazzucchelli R, Lopez-Beltran A, Scarpelli M, Cheng L (2011). Prostatic intraepithelial neoplasia: its morphological and molecular diagnosis and clinical significance. BJU international.

[23] Shah RB, Zhou M (2012). Atypical cribriform lesions of the prostate: clinical significance, differential diagnosis and current concept of intraductal carcinoma of the prostate. Advances in anatomic pathology.

[24] Guo CC, Epstein JI (2006). Intraductal carcinoma of the prostate on needle biopsy: Histologic features and clinical significance. Mod Pathol.

[25] Herawi M, Epstein JI (2007). Immunohistochemical antibody cocktail staining (p63/HMWCK/AMACR) of ductal adenocarcinoma and Gleason pattern 4 cribriform and noncribriform acinar adenocarcinomas of the prostate. The American journal of surgical pathology.

[26] Signoretti S (2000). p63 is a prostate basal cell marker and is required for prostate development. The American journal of pathology.

[27] Wojno KJ, Epstein JI (1995). The utility of basal cell-specific anti-cytokeratin antibody (34 beta E12) in the diagnosis of prostate cancer. A review of 228 cases. The American journal of surgical pathology.

[28] Rubin MA (2002). alpha-Methylacyl coenzyme A racemase as a tissue biomarker for prostate cancer. Jama.

[29] Luo J (2002). Alpha-methylacyl-CoA racemase: a new molecular marker for prostate cancer. Cancer research.

[30] Ng VW, Koh M, Tan SY, Tan PH (2007). Is triple immunostaining with 34betaE12, p63, and racemase in prostate cancer advantageous? A tissue microarray study. American journal of clinical pathology.

[31] Rizzardi AE (2014). Evaluation of protein biomarkers of prostate cancer aggressiveness. BMC cancer.

[32] Rizzardi AE (2012). Quantitative comparison of immunohistochemical staining measured by digital image analysis versus pathologist visual scoring. Diagnostic pathology.

[33] Metzger, G. J., Schmechel, S. C., Dankbar, S. C. & Henriksen, J. Computerized methods for tissue analysis. USA patent US8718350B2 (2012).

[34] Rizzardi AE (2014). Elevated HA and HMMR are associated with biochemical failure in patients with intermediate grade prostate tumors. Cancer.

[35] Pedregosa F (2011). Scikit-learn: Machine Learning in Python. Journal of Machine Learning Research.

[36] Epstein JI (2016). A Contemporary Prostate Cancer Grading System: A Validated Alternative to the Gleason Score. European urology.

[37] Dabir PD, Ottosen P, Hoyer S, Hamilton-Dutoit S (2012). Comparative analysis of three- and two-antibody cocktails to AMACR and basal cell markers for the immunohistochemical diagnosis of prostate carcinoma. Diagnostic pathology.

[38] Kuefer R (2002). alpha-Methylacyl-CoA racemase: expression levels of this novel cancer biomarker depend on tumor differentiation. The American journal of pathology.

[39] Macenko, M. *et al*. In *IE*EE Inte*rnational Symposium on Biomedical Imaging: From Nano to Macro*. 1107–1110.(2009)

[40] van der Loos CM (2008). Multiple immunoenzyme staining: methods and visualizations for the observation with spectral imaging. Journal of Histochemistry and Cytochemistry.

[41] Glass G, Papin JA, Mandell JW (2009). SIMPLE: a sequential immunoperoxidase labeling and erasing method. Journal of Histochemistry and Cytochemistry.

[42] Lotan TL (2013). Cytoplasmic PTEN protein loss distinguishes intraductal carcinoma of the prostate from high-grade prostatic intraepithelial neoplasia. Mod Pathol.

